# Pilot-Testing of “Healthy Body Healthy Mind”: An Integrative Lifestyle Program for Patients With a Mental Illness and Co-morbid Metabolic Syndrome

**DOI:** 10.3389/fpsyt.2019.00091

**Published:** 2019-03-06

**Authors:** Jenifer A. Murphy, Georgina Oliver, Chee H. Ng, Clinton Wain, Jennifer Magennis, Rachelle S. Opie, Amy Bannatyne, Jerome Sarris

**Affiliations:** ^1^Professorial Unit, The Melbourne Clinic, Department of Psychiatry, The University of Melbourne, Melbourne, VIC, Australia; ^2^The Melbourne Clinic, Melbourne, VIC, Australia; ^3^Institute for Physical Activity and Nutrition, School of Exercise and Nutrition Sciences, Deakin University, Geelong, VIC, Australia; ^4^Faculty of Health Sciences and Medicine, Bond University, Gold Coast, QLD, Australia; ^5^NICM Health Research Institute, Western Sydney University, Westmead, NSW, Australia

**Keywords:** healthy lifestyles, lifestyle medicine, metabolic syndrome, depression, program development

## Abstract

**Background:** Metabolic syndrome and co-morbid physical health conditions are highly prevalent in people with a mental illness. Modifiable lifestyle factors have been targeted to improve health outcomes. Healthy Body Healthy Mind (HBHM) program was developed to provide an integrated evidence-based program incorporating practical diet and exercise instruction; alongside meditation and mindfulness strategies, and comprehensive psychoeducation, to improve the physical and mental health of those with a mental illness.

**Methods:** We report on two data points: (1) Qualitative data derived from the first HBHM program (version 1) exploring its utility and acceptance according to patient feedback; (2) Biometric and mental health data collected on the modified and enhanced 12-week HBHM program (version 2) involving a pilot of 10 participants. Mental and physical health outcomes, weight, abdominal circumference, fasting glucose, cholesterol, and triglycerides were measured at program entry and completion.

**Results:** Qualitative data from HBHM version 1 provided valuable feedback to redevelop and enhance the program. At the end of the HBHM (version 2) 12-week program, a significant mean weight loss of 2 kg was achieved, *p* = 0.023. There was also a significant reduction in abdominal circumference (mean = 2.55 cm) and a decrease in BMI of almost one point (mean = 0.96 kg/m^2^), *p* = 0.046 and *p* = 0.019, respectively. There were no significant changes in mental health measures or on any other biometrics.

**Conclusion:** Pilot data from the HBHM program found significant reductions in weight and abdominal obesity. The HBHM program could benefit from further modifications, and study replication is required using a controlled design in a larger sample.

## Introduction

A highly complex relationship exists between physical and mental health. The rates of physical morbidity and mortality in people with mental illness is substantially higher than the general population (1, 2). Even in countries with adequate health care systems, the mortality gap is on the rise, up to a difference of 20 years (3). Obesity and related conditions such as cardiovascular disease, hyperlipidaemia, and diabetes mellitus are highly prevalent in people with mental illness (4).

The cluster of symptoms associated with these lifestyle diseases such as, excess visceral fat, along with hypertension, glucose intolerance/insulin resistance, and/or hyperlipidaemia has been labeled Metabolic Syndrome (MetS). In mental health populations, the rate of MetS is thought to be between 41 and 67% (5). A large proportion of the physical health burden in patients with a mental illness can be linked to side effects of second generation antipsychotics, lifestyle factors (e.g., higher rates of smoking, low levels of exercise), and factors associated with socioeconomic disadvantage (e.g., poverty and lack of access to nutritious foods) (6). Weight gain can result in lowered self-esteem, impaired body-image, and reduced social interactions, which can ultimately lead to poorer mental health (7). Furthermore, it is also a common cause of medication non-adherence, which can lead to illness relapse, hospitalization, and poorer outcomes (7, 8).

There is potential to modify lifestyle factors that contribute to MetS (e.g., caloric intake, physical activity levels, sleep hygiene) to improve the health and well-being of people with a mental illness. There are calls for an integrated response involving early diagnosis, treatment and management of physical and mental health via lifestyle modification (9). Several lifestyle-based programs have been developed to modify the lifestyle factors contributing to MetS (10). These programs typically focus on exercise and nutrition for metabolic issues and result in minimal weight loss and modest effects on well-being (10). In one review of lifestyle interventions in adults with a serious mental illness at risk of MetS, few programs incorporated behavioral components such as stress management and motivation alongside exercise and/or nutrition interventions (10). Thus, programs that target both mental and physical outcomes in patients who are taking psychotropic medication have not been adequately developed. Further, previous programs often do not provide a truly “integrative” model, tending to focus on nutrition and exercise without incorporating components that may improve general health and well-being (10).

To bridge the gap and provide an evidence-based integrated health approach, we developed the “Healthy Body, Healthy Mind Program (HBHM).” The aim was to assist people with a diagnosed mental illness and metabolic syndrome to improve mental and physical well-being. We presently report on: (1) Qualitative data derived from our first version of the HBHM program in 2011, exploring its utility and acceptance according to patient and clinician feedback; (2) Biometric data and mental health data collected on the modified and enhanced 12-week, second version of HBHM program involving a pilot of 10 participants.

## Materials and Methods

### Setting And Overview

The Melbourne Clinic in Melbourne, Australia is a private psychiatric hospital with inpatient and outpatient services. The clinic had a longstanding weight management and lifestyle program, called Healthy Body Healthy Mind (HBHM), developed and facilitated by a general practitioner (GP), exercise physiologist and dietitian. HBHM program (version 1) provided weekly nutritional support from a dietitian, psychoeducation sessions on lifestyle modification and exercise practicals with an exercise physiologist. In late 2011, ethics approval was granted by The Melbourne Clinic Research Ethics Committee (HREC project number: 209) to collect qualitative data from participants to evaluate the perceived efficacy of the program's content, barriers in implementing the components, and feedback on how it could be improved. The qualitative data collected during 2011–2012 was used to redevelop and enhance the integrated lifestyle program which was pilot-tested at The Melbourne Clinic in 2016 (HREC project number: 249). This modified second program will henceforth be labeled as HBHM program (version 2).

### Recruitment

Patients attending The Melbourne Clinic outpatient services were invited to attend the program via a doctor's referral. Patients with a diagnosed mental illness (e.g., Major Depressive Disorder, Bipolar Disorder; or with multiple diagnoses) taking stable psychotropic medication (same dose for at least 4 weeks) with co-morbid diagnosed metabolic syndrome (see Table 1) or obesity (defined as a BMI of >30 OR abdominal circumference of >80 cm for women and >94 cm for men) were the target participants for the program. Written informed consent was obtained from all participants to evaluate the two versions of the program.

**Table 1:** Metabolic syndrome clinical diagnosis guidelines^*^.

**Measure**	**Categorical cut point**
Elevated waist circumference (European/North American)	≥ 102 cm for men, ≥ 88 cm for women
Elevated triglyceride levels (or drug treatment for elevated triglycerides)	≥1.7 mmol/L
Reduced HDL-C (or drug treatment for reduced HDL-C)	< 1.0 mmol/L in men, < 1.3 mmol/L in women
Elevated blood pressure (or drug treatment for hypertension)	≥130 systolic or ≥85 diastolic
Elevated fasting glucose (or drug treatment for elevated glucose)	>5.5 mmol/L

For a diagnosis of Metabolic Syndrome, an individual must meet three of the five specified criteria.

* *As per the International Diabetes Federation Task Force on Epidemiology and Prevention; National Heart Lunch and Blood Institute; American Heart Association; World Heart Federation; International Atherosclerosis Society; and International Association for the Study of Obesity (11)*.

### HBHM Version 2 (2016) Measures

At week 0, a range of medical and demographic information was collected including, age, gender, employment status, medication information, psychiatric diagnosis and medical co-morbidity. At week 0 and week 12, participants also had anthropometric measurements taken by an exercise physiologist (BP, height, weight, abdominal circumference) and completed self-report questionnaires on their mental health (Depression Anxiety Stress Scales—DASS-21). In addition at week 0, participants were asked to state their goals for the program and what they hoped to achieve. They were also asked to rate their readiness to change in order to achieve their listed goals on a scale of 1–10 with 1 being “not ready” and 10 being “ready.” Participants were provided with a blood test request form to have fasting cholesterol, glucose and triglycerides measured at week 0 and week 12. The blood tests were conducted by Australian Clinical Labs.

#### Depression Anxiety Stress Scales—DASS-21

The DASS-21 is a self-reported screening instrument used to measure depression, anxiety and stress symptomatology (12). It consists of 21-items, across three scales, which measure symptoms of depression (7 items), anxiety (7 items), and stress (7 items). Individuals are asked to respond to each item on a four-point Likert scale (0 = *not at all* to 3 = *very much or most of the time*). Higher scores on each scale are indicative of greater symptom severity. Scores above 11 (depression), 8 (anxiety), and 13 (stress) are considered severe, as indicated by the recommended cut-offs (12).

### Statistics

Qualitative data assessing the 2011 HBHM Program (version 1) was analyzed from written participant feedback forms. Comments were coded and grouped in categories by theme (beneficial/useful content, barriers to implementation and program improvements) and counted to obtain a frequency of mention by the 12 participants. For the quantitative data from the 2016 HBHM program (version 2), paired *t*-tests and Pearson's r correlations were used to compare the repeated measurements of the sample (week 0 vs. week 12). All data was analyzed in SPSS 24. An alpha level of < 0.05 was used to determine significance.

## Results

### HBHM Version 1 (2011)—Qualitative Data

Written qualitative data from 12 participants (83% female; mean age 43.4 years old) who participated in the first version of the HBHM program during 2011–2012 was thematically analyzed to aid the development of the 2016 HBHM program. Table 2 summaries the qualitative data on the early program.

**Table 2:** Qualitative feedback from HBHM (version 1).

Beneficial/useful content	Cooking practicals Hands-on experience Meal planning with simple and practical recipes Templates for shopping/organizing pantry Providing alternative foods to swap out “bad” foods Label reading and understanding ratios of good fats, proteins and hidden sugars More understanding of the psychological underpinnings on “why I eat” and overeating Exercise class Chair exercises Fresh air/park exercises Information about benefits of varying exercise Further knowledge on Certain foods and their effects on the body (e.g., what sugar does to the body, proteins, good fats etc.) The harms of poor eating and no exercise on body mechanisms Cravings and how to beat them Reminders of program schedule and encouragement to attend Sharing experiences and “wins” with peers
Barriers	Unable to concentrate/absorb content/participate fully because of low mood/anxiety Injury/pain and physical health issues make exercise difficult or impossible Poor motivation/discipline and difficulty getting started and maintaining momentum Fear of change or not ready to make changes Cooking for one person too hard or can't be bothered Changing the eating patterns of the entire household
Program lessons	Provide notes at each session rather than at the following week Provide diagrams of exercises to do at home Not enough practical cooking/exercise classes Group size too big with new people coming in every week

The most beneficial/useful components of the program reported by the majority of participants were the cooking and exercise practicals (*n* = 11; 91%). One participant commented that he/she needed “*practical examples that can be implemented in day-to-day life”* and less theory in order to engage with the program. However, many participants (*n* = 9; 75%) commented that furthering their knowledge on how food effects the body and the harms of nutritionally poor food and no exercise on the body's functioning was useful as, “*understanding aids motivation to change, oversimplification does not.”* Poor motivation (*n* = 4; 33%), fear of change (*n* = 3; 25%) and difficulties engaging with the program due to low mood and anxiety (*n* = 8; 66.7%) were common among participants. Additionally, many participants found cooking for one person and/or maintaining momentum on their own difficult (*n* = 4; 33%). As one person stated, “*[the] desire to try a new recipe is high at time of session but then lost when at home.”* Participants requested more diagram-based notes of exercises/recipes and program content to be accessible at home due to poor recall (*n* = 3; 25%). Injury and co-morbid physical health problems were some of the main barriers in adding exercise to their day-to-day lives (*n* = 8; 66.67%). At home chair-based exercises and information on how and why to vary exercise was deemed helpful (*n* = 4; 33.3%).

### HBHM Version 2 (2016)—Program Development

Based on the qualitative data detailed above, the HBHM program in 2016 was remodeled and refined in consultation with a team of experts in the fields of psychiatry, endocrinology/cardiology, nutrition and dietetics, and exercise physiology. The program was designed to integrate five evidence-based components: lifestyle psychoeducation (13), exercise (theory and practicals) (14), diet and nutrition (theory and practical skills e.g., cooking demonstrations and label reading exercises) (15), motivation and goal setting skills (16), and mindfulness techniques (17) (see Table 3). Each week, for 12 weeks, participants attended a 6-h session at TMC, where content and practical exercises from each of the five modules was delivered by an exercise physiologist, dietitian and/or a GP (see Table 3 for examples). Participants were provided with a printed booklet each week outlining content for that session (e.g., psychoeducation theory, exercise diagrams, step by step recipes, label reading guidelines) with areas to take notes and complete tasks such as goal setting activities and shopping lists. Table 4 provides an example program day.

**Table 3:** Example HBHM (version 2) practical components and education sessions.

	**Diet and nutrition**	**Exercise**	**Motivation and goal setting**	**Meditation and mindfulness**	**Psychoeducation**
Practical components	- Cooking demonstrations - Excursion to supermarket for healthy shopping tips - Food label reading	- Chair based exercises to increase strength - Pilates - Yoga and stretching - Body weight exercises - Interval, circuit and resistance training	- Goal setting - Ongoing review of personal goals throughout the program	- Meditation to improve sleep - Mindfulness for reducing and managing stress - Mindfulness for self-compassion	N.A
Education session topics	- Why diets don't work? - Sugar—myths and realities - Exploring macronutrients in foods—protein, fats and carbohydrates	- Benefits of physical exercise - The benefits of “greenexercise” (exercising in nature) - Incidental exercise strategies for everyday life	- Ways to plan realistic and attainable goals—SMART goal setting - Finding Motivation—the motivation matrix - Dealing with setbacks and the transtheoretical model of change	- What are the benefits of mindfulness meditation? - Mindful eating for weight loss and well-being	- What is the link between mental and cardiovascular health? - The “brain-gut” connection and its influence on mental & cardiovascular health The importance of sleep and how to improve sleep quality for our health & well-being

**Table 4:** An example HBHM (version 2) program day.

**Time**	**Key component**
9.30–10.00 a.m.	Greeting and brief overview of the session • Includes questions about the previous week or any updates
10.00–10.45 a.m.	Exercise theory and practice with an Exercise physiologist • Education session: Yoga theory and benefits • Practical component: Yoga
10.45–11.00 a.m.	Tea
11.00–12.15 p.m.	Lifestyle psychoeducation with dietitian/GP • Education session: Cortisol—The “good” and “bad” of stress • Brainstorming strategies to manage stress
12.15–1.00 p.m.	Lunch • Practical component: Cooking demonstration with dietitian (example recipe: stuffed capsicums, roast vegetable and grain salad)
1.00–2.00 p.m.	Nutrition theory with dietitian • The evidence, benefits and “how to” of a Mediterranean diet
2.00–2.15 p.m.	Tea
2.15–2.45 p.m.	Mindfulness meditation • Practical session: Mindfulness based stress reduction
2.45–3.30 p.m.	Motivation and goal setting – • Identify goal for the week using SMART guidelines

### HBHM Version 2 (2016)—Pilot Data

#### Characteristics

Fifteen patients were referred to the pilot program, five patients declined to participate and ten patients were willing to take part. All participants attended the week 0 and week 12 sessions. Some of the weekly follow-up sessions were missed due to illness or other personal commitments with an average attendance rate of 80%.

Most of the sample was female (*n* = 8; 80%) with a mean age of 51.8 years (SD = 12.9 years) (see Table 5). Over half the sample were unemployed/not working or on a disability pension (*n* = 6; 60%). The most common primary DSM-IV psychiatric diagnosis as reported in the treating psychiatrist referral to the program was Major Depressive Disorder (MDD) (*n* = 6; 60%) followed by Bipolar Affective Disorder (*n* = 2; 20%), Generalized Anxiety Disorder (GAD) (*n* = 1; 10%) and Alcohol Abuse (*n* = 1; 10%). Psychiatric co-morbidity was common with diagnoses of Generalized Anxiety Disorder (GAD) (*n* = 3; 30%), Binge Eating Disorder (*n* = 1; 10%), Borderline Personality Disorder (*n* = 1; 10%) and Major Depressive Disorder (*n* = 1; 10%).

**Table 5:** HBHM (version 2) Baseline characteristics (*n* = 10).

**Characteristic**	**Mean ± SD/ *n* (%)**
Age	51.80 ± 12.93
Female	8 (80%)
Primary psychiatric diagnosis	
Major Depressive Disorder	6 (60%)
Bipolar Affective Disorder	2 (20%)
Generalized Anxiety Disorder	1 (10%)
Alcohol Dependence	1 (10%)
Primary psychotropic medication^*^	
Antidepressant	9 (90%)
Mood stabilizer	1 (10%)
Other psychotropic medications^*^	
Second generation antipsychotic	6 (60%)
Mood stabilizer	4 (40%)
Antidepressant	2 (20%)
Benzodiazepine	2 (20%)

* *Participants could be taking more than one psychotropic medication (mean = 2.4; SD = 1.17)*.

All participants were taking psychotropic medications defined as an antidepressant, mood stabilizer, second generation antipsychotic or benzodiazepine. The participant's primary medications were agomelatine (*n* = 2; 25–50 mg/day), desvenlafaxine (*n* = 2; 50–100 mg/day), fluvoxamine (*n* = 2; 200–300 mg/day), paroxetine (*n* = 1; 20 mg), venlafaxine (*n* = 1; 75 mg/day); escitalopram (*n* = 1; 40 mg/day); lamotrigine (*n* = 1; 50 mg). On average participants were taking 2.4 (SD = 1.17) psychotropic medications ranging from 1 to 4 psychotropic medications. The most common psychotropic medication taken alongside a primary psychotropic medication were second generation antipsychotics (e.g., quetiapine, lurasidone; *n* = 6; 60%) followed by mood stabilizers (*n* = 4; 40) (see Table 5).

The most common chronic medical conditions self-reported by participants or as listed in the referral to the program were hyperlipidaemia (*n* = 4; 40%), hypertension (*n* = 3; 30%), arthritis (*n* = 3; 30%), Type II Diabetes (*n* = 3; 20%), sleep apnoea (*n* = 2; 20%), and chronic pain/fibromyalgia (*n* = 2; 20%). Other conditions such as, fatty liver disease (*n* = 1; 10%), pancreatitis (*n* = 1; 10%), ulcerative colitis (*n* = 1; 10%), costochondritis (*n* = 1; 10%), fructose malabsorption syndrome (*n* = 1; 10%), endometriosis (*n* = 1; 10%), restless leg syndrome (*n* = 1; 10%) and hypothyroidism (*n* = 1; 10%) wereself-reported.

Table 6 presents the anthropometrics of the participants at week 0 and week 12. The average BMI of the cohort at week 0 was 36.18 (SD = 4.67) which, according to the World Health Organization, classes the participants as Obese Class I. As a cohort, the participants also had impaired glucose tolerance as evidenced by a fasting glucose test average > 5.5 (mean = 5.57 ± 1.19). The participants' cholesterol and BP were normal, most likely due to their use of prescribed statins and hypertensive medications. According to DASS measurement at week 0, participants had low to moderate levels of depression (mean = 8.30; SD = 4.69), anxiety (mean = 6.0; SD = 4.64), and stress (mean = 8.70; SD = 4.24) symptomatology (see Table 6).

**Table 6:** HBHM (version 2) program (*n* = 10) outcome data (week-0 to week-12).

**Outcome**	**Week 0**	**Week 12**	**Paired *t*-test**
Weight (kgs)	100.3 ± 9.79	98.3 ± 9.72	*t* = 2.75 *p* = 0.023^*^
Abdominal circumference (cm)	119.95 ± 12.69	117.40 ± 12.47	*t* = 2.32 *p* = 0.046^*^
BMI	36.18 ± 4.67	35.22 ± 4.73	*t* = 2.86 *p* = 0.019^*^
Waist to hip ratio	0.96 ± 0.08	0.96 ± 0.07	*t* = −0.362 *p* = 0.726
Systolic blood pressure	122.67 ± 15.23	130.25 ± 14.82	*t* = −1.12 *p* = 0.300
Diastolic blood pressure	79.67 ± 11.59	80.88 ± 10.27	*t* = 0.144 *p* = 0.890
Fasting glucose^a^	5.57 ± 1.19	5.72 ± 1.03	*t* = −0.44, *p* = 0.682
HDL cholesterol^a^	1.21 ±.26	1.24 ±.21	*t* = 0.500, *p* = 0.705
LDL cholesterol^a^	2.61 ±.98	3.30 ± 1.20	*t* = −2.00, *p* = 0.295
Total fasting cholesterol^a^	5.34 ± 1.41	5.95 ± 1.20	*t* = −2.61, *p* = 0.080
Triglycerides^a^	2.30 ±.57	2.13 ±.30	*t* = −0.33, *p* = 0.761
DASS 21—Depression score	8.30 ± 4.69	9.70 ± 5.44	*t* = −0.87, *p* = 0.408
DASS 21—Anxiety score	6.00 ± 4.64	5.50 ± 5.10	*t* = 0.75, *p* = 0.475
DASS 21—Stress score	8.70 ± 4.24	8.50 ± 4.72	*t* = 0.38, *p* = 0.716

* *p < 0.05*.

a *Missing data on week 12 (n = 4)*.

#### Participant Goals

The most common goal was to increase exercise levels and fitness (*n* = 5; 50%) followed by eat healthier (*n* = 4; 40%), reduce sugar intake (*n* = 3; 30%) and lose weight (*n* = 3; 30%). On average the participants rated their readiness to change as 7.75 (SD = 2.12) with ratings ranging from 3 to 10. Thus, as a whole the participants were very ready to make change.

#### Outcome Data

The cohort had a significant weight loss over the 12 weeks with an average loss of 2 kg, *t*_(9)_ = 2.75, *p* = 0.023. Weight change ranged from a +1.4 kg weight gain to a −6 kg weight loss. Along with weight loss, there was also a significant reduction in abdominal circumference (mean = 2.55 cm) and a drop in BMI of almost one point (mean = 0.96 kg/m^2^), *t*_(9)_ = 2.32, *p* = 0.046 and *t*_(9)_ = 0.96, *p* = 0.019. No significant changes in participant's blood levels or anthropometrics were found. Additionally, no significant changes in participant's mental health was found on the DASS when comparing week 0 to week 12 (see Table 6). Participant's readiness to change (mean = 7.75; SD = 2.12) was not correlated with change in BMI, abdominal circumference, or weight change at end of the program, *r* = −0.615, *p* = 0.105, *r* = −0.205, *p* = 0.626, and *r* = −0.692, *p* = 0.057, respectively.

## Discussion

This pilot study found that an integrated 12-week lifestyle program was associated with significant weight loss in patients with a mental illness. We found a mean 2 kg weight loss and a drop in BMI of almost one point across the course of 12 weeks. Our findings are in line with a recent meta-analysis which found lifestyle interventions were superior to treatment-as-usual in producing weight loss in patients with a serious mental illness (18). This improvement in anthropometric measures is clinically significant when considering the challenges experienced by patients with a mental illness in maintaining physical and mental wellness.

No beneficial effect on participant's mental health was found despite incorporating meditation, goal setting, mindfulness, and motivation modules, which was a distinguishing feature of the program. This finding was surprising given mindfulness-based therapies have been consistently related to improvements in mood and anxiety in clinical populations (17). However, a recent lifestyle intervention for residential patients with a serious mental illness also found no positive changes on psychosocial outcomes (19). This suggests that it is challenging to get a beneficial effect on psychosocial outcomes while attempting to improve physical and mental health outcomes simultaneously. The addition of individual psychotherapy alongside participation in the program might be warranted and the incorporation of other evidence-based psychotherapies such as, cognitive behavioral therapy should be considered in future versions of the program. Future iterations of the program should also incorporate quality of life assessments and measures of psychosocial functioning to more accurately assess the program's potential benefit.

Alternatively, the modest effect on participant's mental health during the program could be due to the low baseline levels of depression, anxiety, and stress (as captured on the DASS), and hence had less scope for improvement. Furthermore, when identifying goals of the program, not one participant mentioned they would like to improve their mental health. Rather the goals reported by participants were orientated toward improving their eating habits and losing weight. The incorporation of behavioral and psychological components (such as mindful eating and goal setting) is deemed to benefit the delivery and engagement of the program contents and helped to adhere to recommendations in order to achieve the weight loss results.

A major limitation to the current study was the small sample size and that it was uncontrolled. A larger sample with an appropriate control is required to assess the program effectiveness and allow for further feedback from participants to fine-tune the program. We also recognize that more work is needed to refine the delivery and format structure in respect to determining the best application of the program given costs and time constraints. In particular, the exercise component should aim to provide at least 90 min of moderate-to-vigorous exercise per week, as this intensity has been shown to improve psychiatric symptoms in patients with a serious mental illness (20). Furthermore, closer monitoring of physical activity outside of the program should be employed in order to assess the translational impact of the program to the home environment.

While the 12-week program was comprehensive, it also has its drawback in terms of time requirements, and a 6-h once per week delivery is quite intensive for people with a mental illness. A follow-up should also be employed to determine whether weight loss results are maintained, continued, or regained following the cessation of the program.

There is the potential to restructure the program to have a blend of face-to-face delivery with online components, and even the use of a mobile phone app. This would provide flexibility, less demands on time, and less financial burden for the participants. Weight loss in this population is extremely challenging as many patients have co-morbid medical conditions, and limited social and economic resources. Thus, facilitating weight loss using strategies that can be implemented into everyday life is paramount to success. In addition, implementing and assessing strategies which prevent the all too frequent “weight regain” phenomena is crucial (21).

As 60% of the sample were taking second generation antipsychotics, the results are encouraging for metabolically vulnerable patients. However, it is important to note, that the results cannot be generalized to participants with a serious mental illness as the current pilot did not include participants with schizophrenia who are at the most risk of developing MetS. It is unclear how this particular patient population would engage with this intervention. In conclusion, the pilot data of the HBHM program found significant benefits in reducing weight and abdominal obesity, with the potential to achieve substantial health benefits for patients with a mental illness and co-morbid metabolic syndrome. Study replication is required using a controlled design in a larger sample over a longer time period.

## Ethics Statement

This study was carried out in accordance with the recommendations of The National Statement on Ethical Conduct in Human Research 2007 and The Melbourne Clinic Ethics Committee. All subjects gave written informed consent in accordance with the Declaration of Helsinki. The projects were approved by the Melbourne Clinic Ethics Committee (Project 209 and 249).

## Author Contributions

All authors contributed to the drafting and editing of the manuscript. JAM and JS is responsible for the data analysis and manuscript preparation. JM, GO, CW, RO, and AB were involved in designing the program, running the program and collecting data. CN and JS contributed to the design of the program and provided oversight of the program.

### Conflict of Interest Statement

JS has no direct conflict of interest, however has received either presentation honoraria, travel support, clinical trial grants, book royalties, or independent consultancy payments from: Integria Healthcare & MediHerb, Pfizer, Scius Health, Key Pharmaceuticals, Taki Mai, FIT-BioCeuticals, Blackmores, Soho-Flordis, Healthworld, HealthEd, HealthMasters, SPRIM, Kantar Consulting, Research Reviews, Elsevier, Chaminade University, International Society for Affective Disorders, Complementary Medicines Australia, Grunbiotics SPRIM, Terry White Chemists, ANS, Society for Medicinal Plant and Natural Product Research, Sanofi-Aventis, Omega-3 Centre, the National Health and Medical Research Council, CR Roper Fellowship. The funders played no role in the study design, the collection, analysis or interpretation of data, the writing of this paper or the decision to submit it for publication. The remaining authors declare that the research was conducted in the absence of any commercial or financial relationships that could be construed as a potential conflict of interest.
